# Exercise Preserves Lean Mass and Performance during Severe Energy Deficit: The Role of Exercise Volume and Dietary Protein Content

**DOI:** 10.3389/fphys.2017.00483

**Published:** 2017-07-24

**Authors:** Jose A. L. Calbet, Jesús G. Ponce-González, Jaime de La Calle-Herrero, Ismael Perez-Suarez, Marcos Martin-Rincon, Alfredo Santana, David Morales-Alamo, Hans-Christer Holmberg

**Affiliations:** ^1^Department of Physical Education, University of Las Palmas de Gran Canaria Las Palmas de Gran Canaria, Spain; ^2^Research Institute of Biomedical and Health Sciences, Instituto Universitario de Investigaciones Biomédicas y Sanitarias (IUIBS) Las Palmas de Gran Canaria, Spain; ^3^Swedish Winter Sports Research Centre, Department of Health Sciences, Mid Sweden University Östersund, Sweden

**Keywords:** obesity, VLCD, very-low-calorie diet, whey protein, sucrose, exercise

## Abstract

The loss of fat-free mass (FFM) caused by very-low-calorie diets (VLCD) can be attenuated by exercise. The aim of this study was to determine the role played by exercise and dietary protein content in preserving the lean mass and performance of exercised and non-exercised muscles, during a short period of extreme energy deficit (~23 MJ deficit/day). Fifteen overweight men underwent three consecutive experimental phases: baseline assessment (PRE), followed by 4 days of caloric restriction and exercise (CRE) and then 3 days on a control diet combined with reduced exercise (CD). During CRE, the participants ingested a VLCD and performed 45 min of one-arm cranking followed by 8 h walking each day. The VLCD consisted of 0.8 g/kg body weight/day of either whey protein (PRO, *n* = 8) or sucrose (SU, *n* = 7). FFM was reduced after CRE (*P* < 0.001), with the legs and the exercised arm losing proportionally less FFM than the control arm [57% (*P* < 0.05) and 29% (*P* = 0.05), respectively]. Performance during leg pedaling, as reflected by the peak oxygen uptake and power output (Wpeak), was reduced after CRE by 15 and 12%, respectively (*P* < 0.05), and recovered only partially after CD. The deterioration of cycling performance was more pronounced in the whey protein than sucrose group (*P* < 0.05). Wpeak during arm cranking was unchanged in the control arm, but improved in the contralateral arm by arm cranking. There was a linear relationship between the reduction in whole-body FFM between PRE and CRE and the changes in the cortisol/free testosterone ratio (C/FT), serum isoleucine, leucine, tryptophan, valine, BCAA, and EAA (*r* = −0.54 to −0.71, respectively, *P* < 0.05). C/FT tended to be higher in the PRO than the SU group following CRE (*P* = 0.06). In conclusion, concomitant low-intensity exercise such as walking or arm cranking even during an extreme energy deficit results in remarkable preservation of lean mass. The intake of proteins alone may be associated with greater cortisol/free testosterone ratio and is not better than the ingestion of only carbohydrates for preserving FFM and muscle performance in interventions of short duration.

## Introduction

Very low calorie diets (VLCD, <3,350 kJ/day) result not only in loss of fat, but also fat-free mass (FFM; Chaston et al., 2007), mainly due to catabolism of muscle protein to sustain hepatic gluconeogenesis (Cahill, 2006). Excessive loss of FFM may be detrimental, since skeletal muscle accounts for 15–17% of resting metabolism (Muller et al., 2002) and is essential for the preservation of bone mass (Aloia et al., 1995; Vicente-Rodriguez et al., 2005) and exercise capacity (Marks and Rippe, 1996). Furthermore, long-term maintenance of weight is more successful when less FFM is lost during VLCD (Vogels and Westerterp-Plantenga, 2007). For these reasons there is considerable interest in minimizing the loss of FFM during interventions designed to reduce body weight.

Randomized control trials have shown that exercising while following low-calorie diets (LCD; ~4,184 kJ/day normal) attenuates loss of FFM (Janssen and Ross, 1999; Rice et al., 1999; Janssen et al., 2002). It remains unknown whether this effect is limited to the exercised muscles or also affects the non-exercised muscles, as indicated by certain reports (Janssen and Ross, 1999; Janssen et al., 2002). Although FFM can be also preserved by increasing the ratio of protein to carbohydrate in weight-loss diets (Krieger et al., 2006), this effect has only been clearly demonstrated in connection with prolonged interventions involving a moderate energy deficit (Johnston et al., 2004), and appears to depend on the daily intake of protein (Pasiakos et al., 2013). During 2–4 weeks of LCD, combining resistance training or high-intensity interval training with a higher protein intake is more efficient in preserving lean mass and promoting fat mass lost that a control diet with lower daily protein intake (Mettler et al., 2010; Longland et al., 2016). In contrast with these results, lean body mass was reduced similarly in infantry cadets consuming 0.5 or 0.9 g/kg/day dietary protein during 8 days of energy restriction, arduous work, and sleep deprivation causing a daily energy deficit of ~9,600 kJ/day. However, it remains to be determined whether low intensity exercise can preserve lean mass in humans when the energy deficit is as severe as during ironman triathlon competitions (Kimber et al., 2002).

Therefore, our aim was to characterize the potential influence of low-intensity exercise and/or protein ingestion on lean mass during severe energy deficit. For this purpose 15 volunteers were randomly assigned to receive 0.8 g/kg body weight/day of either whey protein (i.e., an amount able to elicit maximal stimulation of protein synthesis; Breen et al., 2011; Witard et al., 2014) or a similar amount of calories in the form of sucrose, during 4 days of extreme energy deficit. Our hypothesis was that a diet consisting solely of whey protein will attenuate the loss of muscle mass and that this effect will be enhanced locally by exercise in a dose-dependent manner, and modulated by the changes in plasma levels of cortisol, testosterone, and amino acids. This hypothesis is based on the fact that exercised muscles are more sensitive to the anabolic effects of circulating amino acids (Apro and Blomstrand, 2010) and testosterone (Bhasin et al., 1996), while being more resistant to the atrophying effects of cortisol (Crowley and Matt, 1996; Krug et al., 2016).

## Methods

### Participants

A full description of our study population and general procedures has been published previously (Calbet et al., 2015). The general characteristics of the 15 overweight volunteer participants are summarized in Table 1. The subjects were assigned randomly to ingest a diet consisting solely of sucrose (SU; *n* = 7) or whey protein (*n* = 8) during the phase of caloric restriction. A study population of this size should reveal any significant difference ≥1.5-fold as large as the coefficient of variation (which was <10% in most cases) between the mean values for any individual parameter, with a significance level of *P* < 0.05 and statistical power of 0.8. This study was pre-approved by the Regional Ethical Review Board of Umeå University (Umeå, Sweden), as well as the ethical committee of the University of Las Palmas de Gran Canaria (Canary Islands, Spain), and after being informed about potential risks and benefits, the participants provided their written consent.

**Table 1:** The baseline characteristics of our subjects.

	**Diet**	
	**Sucrose (*n* = 7)**	**Whey protein (*n* = 8)**
Age (years)	38.7 ± 8.2	43.0 ± 8.0
Height (cm)	181 ± 5.5	180 ± 4.2
Weight (Kg)	98 ± 12.0	100 ± 14.9
BMI (Kg/m^2^)	29.9 ± 3.1	30.9 ± 4.2
Lean mass (Kg)	63.1 ± 3.1	65.4 ± 6.0
Fat mass (Kg)	31.5 ± 9.1	31.4 ± 9.2
Body Fat (%)	31.6 ± 5.3	30.9 ± 4.1
VO_2_max (L/min)	3.8 ± 0.3	3.9 ± 0.3
Physical activity (IPAQ) (kJ/d)	2, 161 ± 1, 318	2, 515 ± 1, 209

*IPAQ, International Physical Activity Questionnaire (previously published in Calbet et al., 2015); BMI, body mass index. The values presented are means ± standard deviation*.

### Experimental protocol

**Figure 1:**
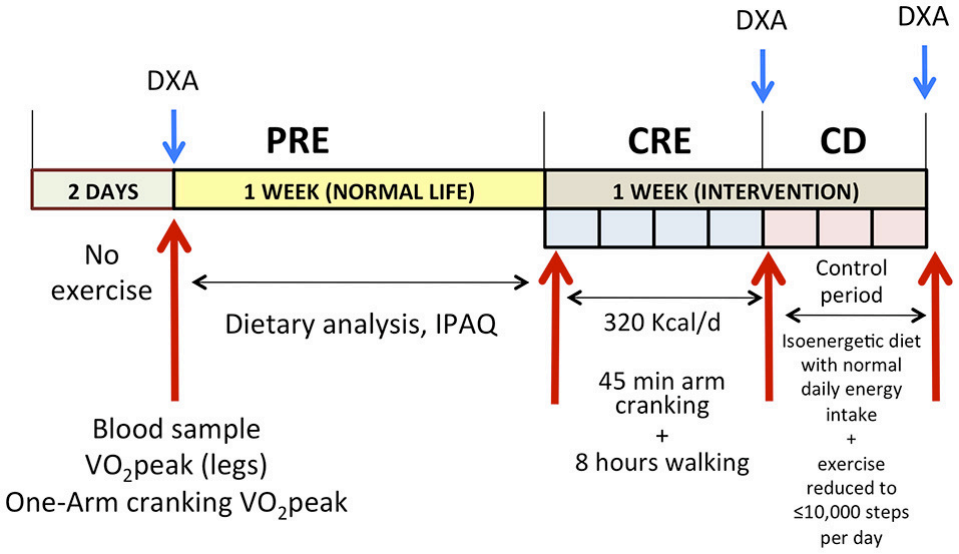
Schematic illustration of the experimental protocol. PRE, baseline tests; CRE, caloric restriction (13.4 kJ/day) and exercise (45 min arm cranking and 8 h walking per day), for 4 days; CD: 3 days on a diet, isoenergetic with that observed during the PRE phase + reduced exercise; DXA, dual-energy x-ray absorptiometry; IPAQ, international physical activity questionnaire.

The protocol involved a baseline phase (PRE), followed by 4 days of caloric restriction and exercise (CRE) and 3 subsequent days on a control diet in combination with reduced exercise (CD; Figure 1). During the PRE and at the end of the CRE and CD phases, body composition (Lunar iDXA, GE Healthcare, Madison, WI, USA) and VO_2_peak (see below) were assessed, and 30-ml blood samples drawn (in the supine position), following a 12-h overnight fast. Although more frequent blood sampling would have allowed circadian rhythms to be taken into account, diurnal variations in cortisol, and testosterone levels are reduced by a prolonged energy deficit (Opstad, 2001), enhancing the representativeness of a single determination.

The PRE phase began with 2 days without exercise, followed by a 12-h overnight fast and drawing a 30-ml blood sample early in the morning of the third day. Another blood sample was taken 7 days later under similar conditions. Since no significant differences were observed between these two samples, their measurements were averaged to obtain a single PRE value. During the 7 days that preceded the CRE phase, the volunteers were asked to eat as usual and record their food intake (see further below). During the first 4 days preceding the CRE phase, baseline body composition, peak oxygen uptake (VO_2_peak) and power (Wpeak) were assessed as described in detail below.

The CRE began with a 12-h overnight fast, after which a blood sample was taken. Each day, the subjects performed 45 min of one-arm cranking at 15% of the Wpeak attained during the incremental one-arm cranking test to exhaustion. The arm with which the 45-min exercise was performed was chosen randomly. Thereafter, the subjects walked during 8 h (at temperatures ranging from 2.9 to 10.2°C) along forest trails and country roads in the vicinity of the Swedish Winter Sports Research Center in Östersund. Each covered 35 km at 4.5 km/h daily (RS800CX Heart Rate Monitor equipped with GPS, Polar Oy, Oulu, Finland), with 5 min of rest every hour. In a double-blind fashion they ingested either sucrose or whey protein (0.8 g/kg body weight) (Syntrax Nectar, Syntrax Innovations, Scott City, MO, USA), both dissolved in 1.5 L water containing minerals, as their sole nutrients, providing an overall energy intake of 13.4 kJ/kg body weight/day (Table 2). The solution of whey protein also contained Na^+^ (308 mg/L) and K^+^ (370 mg/L), as did the sucrose solution (160 mg of Na^+^ and 100 mg of K^+^ per L). The subjects drank 0.5 L of this solution in the morning (just before arm-cranking) and again at midday and 8 PM. Thus, the last “meal” was consumed at the end of the walks, to facilitate overnight protein synthesis in those ingesting whey protein (Snijders et al., 2015). In addition, both groups were allowed to drink a hypotonic rehydrating solution containing Na^+^ (160 mg/L), Cl^−^ (200 mg/L), K^+^ (100 mg/L), citrate (700 mg/L), and sucrose (3 g/L) *ad libitum*. Despite the difference in the composition of these drinks, the sucrose and whey protein groups drank similar amounts of rehydrating solution daily [3.6 ± 0.9 and 3.3 ± 0.7 L/day (*P* = 0.51) as well as 10.7 ± 2.7 and 9.9 ± 2.2 g/day of additional sucrose (*P* = 0.51), respectively] during the CRE phase.

**Table 2:** Composition of the diets during the three different phases of this study.

		**Diet**	
		**Sucrose (*n* = 7)**	**Whey protein (*n* = 8)**
PRE	Carbohydrate (g)	211.5 ± 59.4	212.3 ± 59.8
	Fat (g)	92.0 ± 14.6	80.2 ± 21.7
	Protein (g)	99.8 ± 29.6	83.7 ± 18.0
	Fiber (g)	18.7 ± 5.3	17.1 ± 4.5
	Ethanol (g)	26.1 ± 17.4	25.7 ± 14.2
	Energy (kJ)	9, 439 ± 2, 145	8, 728 ± 2, 047
CRE	Carbohydrate (g)	89.1 ± 11.4	9.9 ± 2.2
	Fat (g)	0.0	0.0
	Protein (g)	0.0	80.1 ± 11.9
	Fiber (g)	0.0	0.0
	Ethanol (g)	0.0	0.0
	Energy (kJ)	1, 492 ± 191	1, 506 ± 215
CD	Carbohydrate (g)	265.7 ± 46.1	265.8 ± 48.1
	Fat (g)	90.8 ± 17.2	90.6 ± 11.9
	Protein (g)	71.1 ± 14.2	70.7 ± 10.4
	Fiber (g)	36.7 ± 9.3	35.1 ± 5.6
	Ethanol (g)	0.0	0.0
	Energy (kJ)	8, 936 ± 1, 632	8, 907 ± 1, 398

*PRE, baseline measurements; CRE, 4 days of caloric restriction and exercise; CD, 3 days of control diet combined with reduced exercise (CD). The values presented are means ± standard deviation*.

Each day during the CD phase, each participant ate three standardized meals containing his normal daily intake of energy (as assessed by weighing all food ingested during 7 days of the pre-test period) and was not allowed to walk more than 10,000 steps. This phase was designed to allow replenishment of body water and stabilization of body weight.

During CRE and CD, participants went to bed around 22.00 h and woke-up around 06.30 h.

### Assessment of physical activity, nutrition, and body composition

The short version of the International Physical Activity Questionnaire was employed to assess daily energy expenditure related to physical activity (Craig et al., 2003; Serrano-Sanchez et al., 2010). During 7 days of the PRE phase each participant kept a dietary record and his food was analyzed (Dietist XP, Kost & Näringsdata, Bromma, Sweden). During CD, all were provided a diet with the same energy content as that recorded during PRE and the weight of the food they ingested determined. Energy intake was also calculated employing the Dietist XP program. During the PRE phase, the sucrose and whey protein groups ingested 9,439 ± 2,145 and 8,728 ± 2,047 kJ/d (means ± *SD*), respectively. During CD, the corresponding values were 8,936 ± 1,632 and 8,907 ± 1,398 kJ/d, with the protein component of the diet representing 0.76 and 0.75 g/kg of body weight/day in the sucrose and weight protein groups, respectively (Table 2).

In the morning, following a 12-h overnight fast, body composition was determined by dual-energy x-ray absorptiometry as reported elsewhere (Calbet et al., 1998). Lean mass (LM) was defined as LM = total tissue mass − fat mass − bone mineral content.

### Assessment of Vo_2_peak

Oxygen uptake and CO_2_ production were measured employing a metabolic cart (Jaeger Oxycon Pro, Viasys Healthcare, Hoechberg, Germany), calibrated with 16.0% O_2_ and 4.0% CO_2_ (Air Liquide, Kungsängen, Sweden), at low, medium, and high flow rates utilizing a 3-L air syringe (Hans Rudolph Inc., Kansas City, MO, USA), in accordance with the recommendations of the manufacturer. VO_2_peak values for the whole-body and control and exercised arms were determined by one-arm cranking with the control or exercised arm alone (in random order), followed by two-legged pedaling (Achten et al., 2002; Ponce-Gonzalez et al., 2016). The arm-cranking test began at 10 W for 5 min, followed by a 10-W increase every 3 min. The two-legged pedaling test started at 30 W for 5 min, followed by a 30-W increment every 3 min. At the end of the 3-min period during which the subject exhibited an RER > 1.0, exercise was stopped. Following 5 min of recovery, an incremental test to exhaustion (10 or 30 W/min, for the arm and leg protocols, respectively), beginning at the highest load reached during the previous phase, was performed to determine the VO_2_peak. During the tests the participants were instructed to maintain cranking and pedaling rates of 80 rpm. Between tests, they were allowed to recover long enough for the capillary blood level of lactate (taken from the earlobe) to fall below 3.0 mmol/L (Biosen C-line, EKF Diagnostics GmbH, Barleben, Germany). The highest VO_2_ value during any 20-s interval of an incremental test on the cycle ergometer (Monark Ergomedic 839E, Monark Exercise AB, Vansbro, Sweden) was designated as the VO_2_peak.

### Biochemical and hormonal analyses

After a 12-h overnight fast, 30-mL blood samples were drawn from an antecubital vein directly into Vacutainer Tubes (REF: 368499; 368498). Some samples were collected in tubes containing EDTA and centrifuged for 5 min at 2,000 g and 4°C, to obtain plasma; while others were centrifuged for 10 min at 2,000 g and 4°C to prepare serum. All of these samples were quickly stored at −80°C until being analyzed.

The concentration of glucose in serum was measured with the hexokinase procedure, utilizing kits from ABX Pentra (Horiba Medical, Montpellier, France). Serum insulin was quantified by an electrochemiluminescence immunoassay (ECLIA) performed with reagent kits and a Modular Analytics Analyzer E170 (Roche Diagnostics SL, Barcelona, Spain), at a sensitivity of 0.2 μU/mL and with corresponding intra- and inter-assay coefficients of variation of 2.0 and 2.6%, respectively. Cortisol and total testosterone were measured with chemiluminescence enzyme immunoassays (Immulite 2000 Cortisol, Ref. L2KCO2, Immulite 2000 Total Testosterone, Ref. L2KTW2; Siemens) exhibiting sensitivities of 5.5 and 0.5 nmol/L and intra- and inter-assay coefficients of variation of 6.2 and 7.3%, and 8.2 and 9.1%, respectively. Free testosterone was determined by a radioimmunoassay (Coat-A-Count Free Testosterone, Ref. TKTF1; Siemens) with a sensitivity of 0.5 pmol/L and intra- and inter-assay coefficients of variation of <8%. Sexual hormone-binding globulin (SHBG) was assessed with a chemiluminescence enzyme immunoassay (Immulite SHBG, Ref. L2KSH2; Siemens) characterized by a sensitivity of 0.02 nmol/L and intra- and inter-assay coefficients of variation of 2.7 and 5.2%, respectively. The free androgen index (FAI) was calculated as [TT (nmol/L)/SHBG (nmol/L)] × 100.

Following automated precolumn derivatization of plasma amino acids with o-phthalaldehyde, the resulting derivatives were separated by reversed-phase HPLC (on a 5-μm Resolve C18 column; Waters), and quantified by fluorescence detection. The derivatization reagent was prepared by dissolving 50 mg o-phthalaldehyde in 1 mL of methanol and then adding 9 mL potassium borate buffer (0.4 mol/L, pH 10) and a 50 μL 2-mercaptoethanol. Solvent A consisted of phosphate buffer (0.1 mol/L, pH 7.0)/methanol/tetrahydrofuran (96:2:2) and Solvent B methanol/water (65:35). The HPLC system contained two Model 510 pumps, a PCM Pump Control Module, a WISP 710 autosampler and a Model 470 fluorescence detector, all from Waters (Barcelona, Spain). Measurements were performed with an excitation wavelength of 338 nm and emission wavelength of 425 nm and the data collected and processed by a Model 860 Waters Networking Computer System.

The tryptophan ratio was calculated as the plasma concentration of tryptophan divided by the sum of phenylalanine, tyrosine and the BCAA (Fernstrom and Wurtman, 1972; Fernstrom, 2013).

### Statistical analyses

Values were checked for normal distribution using the Shapiro–Wilks test and, when necessary, transformed logarithmically before the analysis. A repeated-measures ANOVA with time and the two different diets (sucrose vs. whey protein) within-subjects factors was applied for analysis of the amino acids and hormonal mean responses. Lean mass changes were analyzed using repeated-measures ANOVA with time, the two different diets (sucrose vs. whey protein) and three extremities as within-subjects factors. The Mauchly's test of sphericity was run before the ANOVA and in the case of violation of the sphericity assumption, the degrees of freedom were adjusted according to the Huynh and Feldt test. When a significant main effect or interaction was observed, pairwise comparisons at specific time-points were adjusted for multiple comparisons with the Holm–Bonferroni procedure. The relationship between variables was examined by simple and step-wise multiple linear regression. The values reported are means ± standard deviations and a *P* ≤ 0.05 was considered to be statistically significant. All statistical analyses were performed using SPSS v.15.0 for Windows (SPSS Inc., Chicago, IL, USA).

## Results

### Lean mass

**Figure 2:**
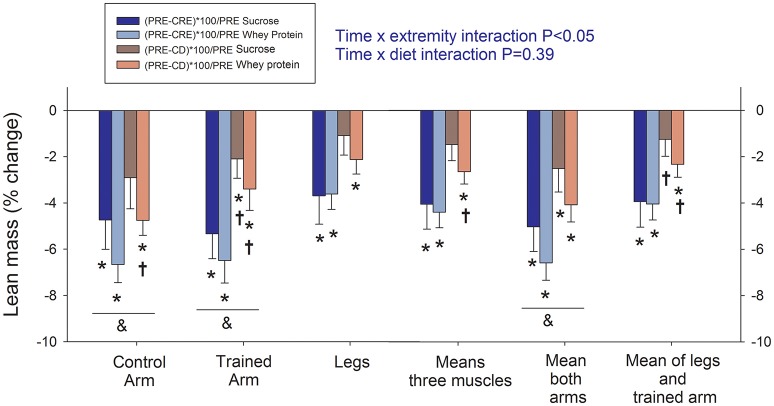
Assessment of changes in lean mass (fat-free mass—bone mass) by dual-energy x-ray absorptiometry. PRE, baseline tests; CRE, caloric restriction (13.4 kJ/day) and exercise (45 min arm cranking and 8 h walking per day) for 4 days; CD: 3 days on a diet isoenergetic with that consumed during the PRE phase + reduced exercise. The vertical bars represent the mean values and the error bars the standard error of the mean. Sucrose: in dark colors (*n* = 7) and whey protein: in light colors (*n* = 8). ^*^P < 0.05 compared to PRE; ^†^*P* < 0.05 compared to CRE; ^&^*P* < 0.05 arms compared to legs.

The major alterations in body composition elicited by the present intervention, as well as the changes in plasma levels of lipids, leptin, cortisol, glucose and insulin have been reported previously (Calbet et al., 2015). Lean body mass was reduced from 64.3 ± 4.9 at PRE to 61.5 ± 4.7 and 63.3 ± 4.5 Kg CRE and CD, respectively (all comparisons *P* < 0.01). Loss of lean mass after CRE was similar in both groups (time × diet interaction *P* = 0.34), but was greater for the arms (~ −6%) than for the legs (~ −4%; time × extremity interaction *P* < 0.05; Figure 2). Despite a mild negative energy balance during the 3 days on the control diet, ~50% of the lean mass lost from the four limbs was recovered as a result of replenishment of water stores, as previously reported (Calbet et al., 2015). However, compared to PRE, the reduction of lean mass at CD was significantly greater for the control arm (−3.9 ± 2.8%) than for the trained arm (−2.8 ± 2.4%, *P* = 0.05) and the legs (1.6 ± 2.0%, *P* < 0.05).

### Peak power output and VO_2_peak during leg pedaling

**Figure 3:**
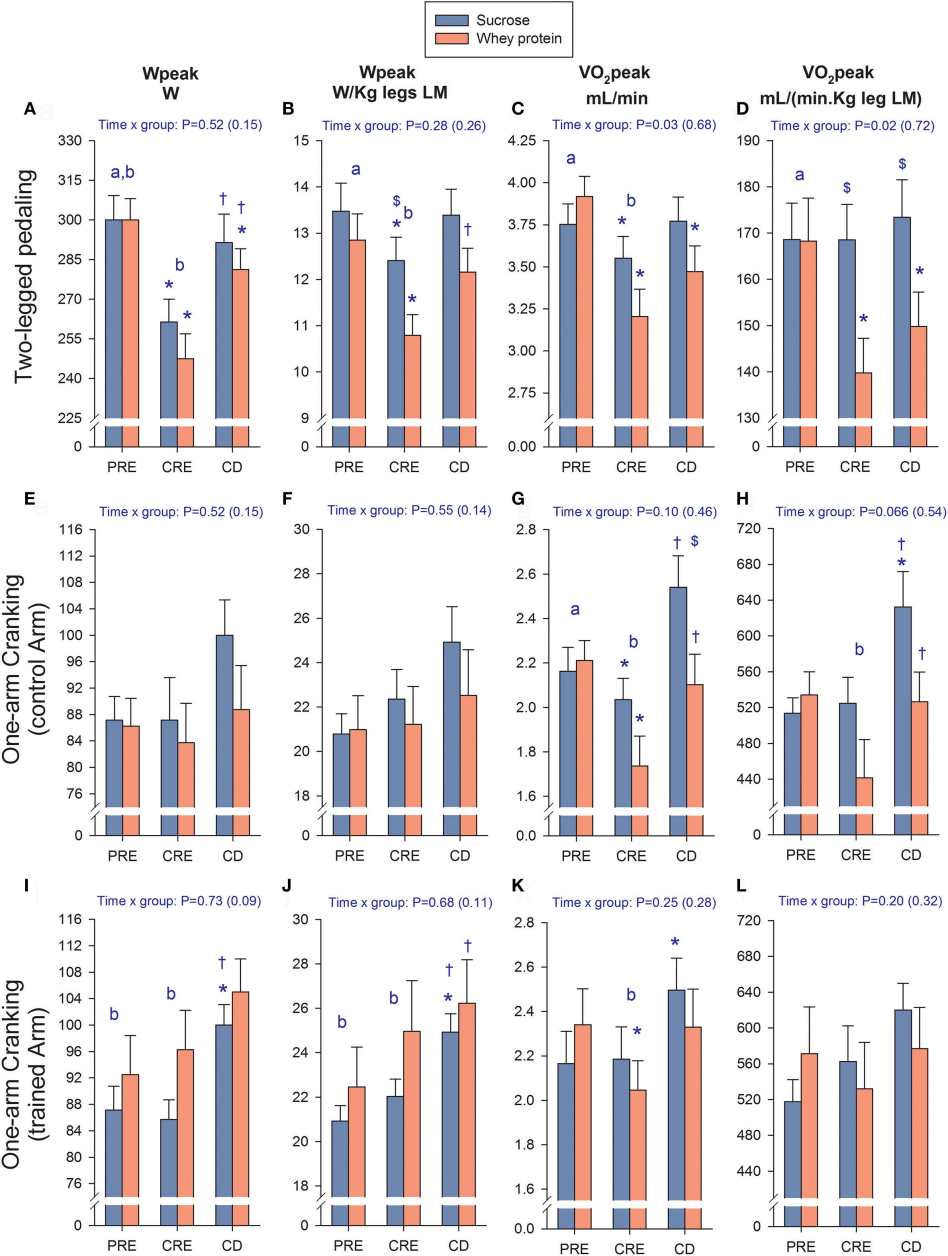
Changes in performance. Assessment of changes in peak power output (Wpeak) and peak oxygen uptake (VO_2_peak) in absolute and relative values (LM, lean mass) during two-legged pedaling **(A–D)**, one-arm cranking with the control arm **(E–H)**, and one-arm cranking with the trained arm **(I–L)**. PRE, baseline tests; CRE, caloric restriction (13.4 kJ/day) and exercise (45 min arm cranking and 8 h walking per day) for 4 days; CD, 3 days on a diet isoenergetic with that consumed during the PRE phase + reduced exercise. The vertical bars represent the mean values and the error bars the standard error of the mean. Sucrose (*n* = 7) and whey protein group (*n* = 8). ^*^P < 0.05 compared to PRE; ^†^*P* < 0.05 compared to CRE; ^$^*P* < 0.05 sucrose compared to whey protein; ^a^*P* < 0.05 compared to CRE (time main effect); ^b^*P* < 0.05 compared to CD (time main effect).

Peak power output (Wpeak) and VO_2_peak after CRE were 15 and 12% lower, respectively, than the corresponding PRE values (300 ± 23 vs. 254 ± 25 W; and 84 ± 0.33 vs. 3.37 ± 0.43 L/min, *P* < 0.01; Figures 3A,C). Following the 3 days on a control diet, performance was improved; however, Wpeak remained 4.7% below the PRE value (286 ± 25 W, *P* = 0.029) and VO_2_peak was still 5.5% lower (3.63 ± 0.43 L/min, *P* = 0.058). These reductions were due partially to the loss of lean mass (LM; Figures 3B,D). From PRE to CRE, the Wpeak (W/kg LM) and VO_2_peak (mL/kg LM.min) for the legs fell 12.2 and 12.3%, respectively (from 13.1 ± 1.6 to 11.5 ± 1.5 W/kg; and from 168 ± 23 to 153 ± 25 mL/(kg.min), *P* < 0.05). Following the 3 days on a control diet the relative Wpeak and VO_2_peak values were 3.1 and 4.5% below PRE levels (both *P* = 0.18). The reduction in Wpeak per kg of LM from PRE to CRE was more pronounced in the whey protein than sucrose group (16.0 and 7.9%, respectively, *P* = 0.051), as was the reduction in VO_2_peak, both in absolute (18.2 and 5.3%, *P* = 0.051) and relative terms (16.9 and 0.0%, *P* = 0.024). After 3 days on the control diet, the absolute Wpeak and VO_2_peak values for the sucrose group were the same as PRE, but remained 6.3 and 11.3% lower, respectively, in the case of the whey protein group (both *P* < 0.05). In relative terms, VO_2_peak had recovered fully in the sucrose group, but remained 11.0% lower in the whey protein group (*P* = 0.03).

### Peak power output and VO_2_peak during one-arm cranking

Training increased both the absolute and relative Wpeak of the exercised, but not the control arm (*P* < 0.05, ANOVA main training effect; Figures 3E,F,I–L), whereas the insignificant alteration in the exercised arm VO_2_peak could be explained by the change in lean mass (Figures 3K,L). From PRE to CRE, the absolute VO_2_peak of the control arm fell 14% (*P* = 0.018), due mainly to a 21% reduction in the protein group (*P* = 0.029; Figures 3G,H), an effect that could be only partially explained by the reduction in lean mass (Figure 3H). After 3 days on the control diet, the VO_2_peak of the control arm was again similar to PRE (*P* = 0.39), or even higher for the sucrose group when the reduction in lean mass was taken into account (*P* = 0.049; Figures 3G,H).

### Metabolic and hormonal changes

As documented in Table 3, although the total plasma levels of the amino acids examined were not altered significantly, exercise and diet affected the levels of certain individual amino acids. The levels of alanine and tryptophan were reduced after CRE and CD, to the same extent in both groups. Concentrations of asparagine, isoleucine, leucine, and valine were higher after CRE, as were the combined levels of the three BCAA (branched-chain amino acids) and the essential amino acids (plus glutamine), with the latter effects being more pronounced in those who ingested whey protein. After CRE, the tryptophan ratio in plasma was reduced to a similar extent (*P* < 0.001) in the sucrose and whey groups (from 0.093 ± 0.016 to 0.063 ± 0.011 and from 0.100 ± 0.010 to 0.059 ± 0.008, respectively, *P* = 0.29).

**Table 3:** Plasma amino acids.

**Amino acids (μmol/L)**	**Group diet during CRE**	**Pre-test (PRE)**	**Exercise + caloric restriction (CRE)**	**Control diet + limited exercise (CD)**	**Time effect**	**Time × diet interaction P (Power)**
Alanine	Sucrose	316 ± 63	246 ± 30	270 ± 26	a, b	0.78 (0.09)
	Whey protein	315 ± 82	249 ± 50	251 ± 33		
Arginine	Sucrose	112 ± 27	97 ± 15	133 ± 35	*P* = 0.22 (0.31)	0.82 (0.08)
	Whey protein	137 ± 34	120 ± 39	124 ± 35		
Asparagine	Sucrose	153 ± 22	184 ± 17	194 ± 34	a, b	0.19 (0.34)
	Whey protein	168 ± 37	192 ± 42	182 ± 32		
Aspartate	Sucrose	8.6 ± 1.3	9.6 ± 1.3	9.4 ± 4.1	*P* = 0.47 (0.17)	0.46 (0.17)
	Whey protein	9.3 ± 2.7	9.9 ± 1.2	8.4 ± 2.0		
Glutamate	Sucrose	81 ± 18	91 ± 115	56 ± 11	*P* = 0.42 (0.12)	0.33 (0.16)
	Whey protein	75 ± 32	52 ± 20	62 ± 23		
	Whey protein	1, 319 ± 185	1, 327 ± 239	1, 325 ± 189		
Glycine	Sucrose	240 ± 26	241 ± 35	256 ± 27	b, c	0.11 (0.44)
	Whey protein	239 ± 41	220 ± 34	277 ± 33^*^		
Isoleucine	Sucrose	91 ± 16	111 ± 9^*^	95 ± 12	a, c	0.001 (0.99)
	Whey protein	88 ± 10	155 ± 32^*^	89 ± 18^†^		
Leucine	Sucrose	162 ± 12	219 ± 18^*^	162 ± 12^†^	a, c	0.001 (0.95)
	Whey protein	150 ± 22	264 ± 44^*^	150 ± 22^†^		
Lysine	Sucrose	145 ± 21	172 ± 34	165 ± 19	c	0.07 (0.53)
	Whey protein	152 ± 29	147 ± 26	180 ± 43		
Methionine	Sucrose	32 ± 5	35 ± 4	32 ± 8	*P* = 0.23 (= 0.30)	0.72 (0.10)
	Whey protein	31 ± 4	38 ± 11	36 ± 10		
Phenylalanine	Sucrose	86 ± 5	73 ± 5^*^	71 ± 8^*^	c	0.04 (0.62)
	Whey protein	77 ± 8	81 ± 15	70 ± 9		
Serine	Sucrose	122 ± 25	125 ± 19	148 ± 19^*^^†^	b, c	0.63 (0.12)
	Whey protein	120 ± 22	116 ± 22	153 ± 34^*^^†^		
Threonine	Sucrose	142 ± 21	147 ± 37	172 ± 29	c	0.08 (0.51)
	Whey protein	164 ± 43	131 ± 34	162 ± 49		
Tryptophan	Sucrose	65 ± 9	49 ± 9	56 ± 10	a, b	0.47 (0.17)
	Whey protein	66 ± 8	59 ± 14	55 ± 15		
Tyrosine	Sucrose	72 ± 13	71 ± 22	72 ± 22	*P* = 0.61 (0.13)	0.49 (0.16)
	Whey protein	61 ± 18	75 ± 15	64 ± 19		
Valine	Sucrose	296 ± 24	314 ± 35	272 ± 25	a, b, c	0.001 (0.99)
	Whey protein	281 ± 26	418 ± 66^*^	251 ± 40^†^		
BCAA	Sucrose	549 ± 33	644 ± 58^*^	525 ± 43^†^	a, b, c	0.001 (0.99)
	Whey protein	518 ± 50	837 ± 136^*^	480 ± 69^†^		
EAA	Sucrose	2, 350 ± 218	2, 437 ± 258	2, 429 ± 188	a, c	0.03 (0.79)
	Whey protein	2, 326 ± 237	2, 618 ± 367^*^	2, 308 ± 285^†^		
TMAA	Sucrose	3, 455 ± 274	3, 502 ± 310	3, 568 ± 280	*P* = 0.21 (0.31)	0.13 (0.41)
	Whey protein	3, 450 ± 414	3, 652 ± 493	3, 428 ± 353		

* P < 0.05 in comparison to PRE;

† *P < 0.05 in comparison to CRE*.

*a, P < 0.05 for PRE vs. CRE; b, P < 0.05 for PRE vs. CD; c, P < 0.05 for CRE vs. CD*.

BCAA, branched chain amino acids; TMAA, total measured amino acids; EAA, essential amino acids (including glutamine). ^*^P < 0.05 in comparison to PRE; ^†^P < 0.05 in comparison to CRE. a, P < 0.05 for PRE vs. CRE; b, P < 0.05 for PRE vs. CD; c, P < 0.05 for CRE vs. CD. The values presented are means ± standard deviation.

Plasma levels of insulin and free testosterone and the FAI were reduced, whereas SHBG, cortisol, and the catabolic index (cortisol/total testosterone and cortisol/free testosterone) were higher after CRE and CD (Table 4). Although there were no significant differences between groups after CRE, the C/FT index tended to be greater in the whey protein than sucrose group (*P* = 0.06).

**Table 4:** Changes in the plasma levels of glucose and hormones.

**Variables**	**Diet during caloric restriction**	**Pre-test (PRE)**	**Exercise + caloric restriction (CRE)**	**Control diet + limited exercise (CD)**	**Time effect**	**Time × diet interaction P (Power)**
Glucose (mmol/L)	Sucrose	5.8 ± 0.4	4.9 ± 0.8^*^	5.2 ± 0.5^*^	a, b, c	0.06 (0.51)
	Whey protein	5.4 ± 0.4	4.6 ± 0.4^*^	5.4 ± 0.3^†^		
Insulin (mUI/L) §	Sucrose	8.5 ± 3.3	4.9 ± 1.8^*^	6.7 ± 1.7^†^	a, b, c	0.82 (0.08)
	Whey protein	12.1 ± 9.9	5.8 ± 2.3^*^	7.4 ± 3.8^*^		
Total Testosterone (nmol/L)	Sucrose	14.0 ± 3.7	9.1 ± 3.5^*^	14.8 ± 4.6^†^	a, c	0.92 (0.06)
	Whey protein	11.7 ± 5.1	7.2 ± 4.2^*^	12.3 ± 5.0^†^		
Free Testosterone (pmol/L)	Sucrose	45.9 ± 11.2	30.5 ± 15.5	38.1 ± 11.1	a, b, c	0.99 (0.05)
	Whey protein	38.2 ± 21.4	22.9 ± 13.4	30.3 ± 12.2		
SHBG (nmol/L)	Sucrose	29.0 ± 10.2	38.5 ± 13.1^*^	41.9 ± 12.6^*^	a, b, c	0.33 (0.23)
	Whey protein	23.1 ± 7.9	29.2 ± 11.1^*^	33.2 ± 11.3^*^^†^		
FAI	Sucrose	0.52 ± 0.19	0.25 ± 0.11^*^	0.37 ± 0.12^*^^†^	a, b, c	0.90 (0.06)
	Whey protein	0.51 ± 0.14	0.24 ± 0.07^*^	0.38 ± 0.12^†^		
Cortisol (nmol/L)	Sucrose	615 ± 97	686 ± 108	660 ± 88	a, b	0.13 (0.42)
	Whey protein	682 ± 86	811 ± 210	643 ± 120^†^		
C/TT §	Sucrose	46.5 ± 13.2	85.4 ± 33.3^*^	49.0 ± 18.1^†^	a, b	0.25 (0.28)
	Whey protein	68.1 ± 27.3	149.6 ± 78.1^*^	60.5 ± 26.8^†^		
C/FT (×10^−3^) §	Sucrose	14.0 ± 2.9	27.4 ± 12.0^*^	18.7 ± 5.9^*^^†^	a, b, c	0.38 (0.21)
	Whey protein	22.5 ± 11.9	48.5 ± 27.2^*^	24.3 ± 10.4^†^		

FAI, Free Androgen Index; SHBG, Sexual Hormone Binding Globulin; C/TT, cortisol/total testosterone; C/FT, Cortisol/ free testosterone.

* P < 0.05 in comparison to PRE;

† *P < 0.05 in comparison to CR+Exercise. a, P < 0.05 for phase PRE vs. phase CRE; b, P < 0.05 for PRE vs. phase CD; c, P < 0.05 for CRE vs. phase CD; § log-transformed. The values presented are means ± standard deviation*.

The difference in whole-body lean mass between PRE and CRE exhibited correlations to the levels of total and free testosterone, the FAI, and the C/FT ratio (*r* = 0.65, 0.61, 0.57, −0.58, respectively, *P* < 0.05). The correlation between the change in lean mass and C/FT index was due primarily to the pronounced association observed between these two in the whey protein group (*r* = −0.82, *P* < 0.05, *n* = 8). The change in the C/FT index was also associated with the change in insulin concentration (*r* = 0.56, *P* < 0.05). Multiple regression analysis showed that only the C/FT index could predict the change in lean mass while the changes in insulin were excluded from the model.

Moreover, there was a linear relationship between the difference in whole-body lean mass between PRE and CRE and the corresponding differences in plasma levels of isoleucine, leucine, tryptophan, valine, BCAA, and EAA (essential amino acids; *r* = −0.58, −0.54, −0.71, −0.59, −0.60, and −0.63, respectively, *P* < 0.05). Similarly, there was a positive linear relationship between the difference in the C/FT ratio and the serum levels of BCAA between PRE and CRE for the group ingesting protein (*r* = 0.70, *P* = 0.05), but not in the case of sucrose (*r* = 0.01, *P* = 0.98).

## Discussion

The ability of dietary protein supplementation and exercise to preserve skeletal muscle mass and function during 4 days of severe energy deficit was examined here. One arm performed 45 min of low-intensity exercise daily (the other arm serving as a “sedentary” control), volume of exercise similar to that employed in many exercise programs (Shaw et al., 2009), while the legs exercised for 8 h daily, an exceptional level of activity. The three muscles examined were exposed to similar levels of circulating amino acids and endocrine signals, but different amounts of exercise. Thus, this experimental design allowed examining whether the catabolic response to a severe daily energy deficit similar to that during ironman triathlon competitions (Kimber et al., 2002), but repeated during 4 consecutive days, affects differently arms and leg muscles. Moreover, by having one group of subjects without protein ingestion, we have been able to test specifically whether the ingestion of protein potentiates the lean mass sparing effect of exercise during VLCD.

Our present findings demonstrate clearly that exercise has a remarkable ability to preserve lean mass, even with an energy deficit of nearly 23 MJ/day, as calculated from the body composition changes (Calbet et al., 2015). As little as 45 min of low-intensity exercise attenuated the loss of arm lean mass by 29% compared to that observed in the non-exercised control arm. Compared to the control arm, 8 h of walking reduced the loss of leg lean mass by 57% indicating that the protective effect of exercise is increased by a more prolonged contractile activity. Nevertheless, maximal exercise capacity was deteriorated only during leg pedaling, as reflected by the lower VO_2_peak and Wpeak values following the severe energy deficit and to a greater extent in the whey protein group than in the sucrose group.

### Changes in performance after a severe energy deficit

Performance (as reflected by Wpeak and VO_2_peak) was deteriorated after the 4 days of exercise with caloric restriction in the legs and control arm, while being maintained in the exercised arm. In agreement with this effect on the legs, Guezennec et al. (1994) found that the VO_2_max of 9 male soldiers was reduced 8% by 5 days of continuous physical activity involving an estimated energy deficit of 13.6 MJ/days.

In the present investigation, the reduction in performance was more pronounced in the whey protein than in the sucrose group, implying that during a severe energy deficit the administration of proteins alone may be less efficient to maintain muscle performance in the legs than the administration of sucrose alone. The reason why exercise together with whey protein prevented the deterioration of VO_2_peak in the exercised arm but not in the legs may be related to differences between extremities in fiber types (Zinner et al., 2016), the volume of exercise, energy metabolism (Van Hall et al., 2003; Zinner et al., 2016) and sensitivity to anabolic/catabolic stimuli (Guerra et al., 2014). Although not measured here, after CRE the level of glycogen in leg muscles could have been reduced to a greater extent than in the arm muscles. However, low muscle glycogen levels *per-se* do not seem to reduce VO_2_peak (Sabapathy et al., 2006). Moreover, after the 4 days of prolonged exercise with caloric restriction, RER values during submaximal exercise on the cycle ergometer were reduced to a similar extent in both groups (not shown). If the muscle level if glycogen had been maintained more effectively in the sucrose group, then this group should have exhibited a higher RER during submaximal exercise (Harger-Domitrovich et al., 2007). Moreover, to attenuate glycogen depletion during prolonged exercise a much greater amount of carbohydrate than the administered here must be ingested (Harger-Domitrovich et al., 2007).

The legs performed an amount of exercise proportionally similar to that carried out by ultra-endurance athletes, which could have damaged mitochondria (Fernstrom et al., 2007; Sahlin et al., 2010) and promoted autophagy and mitophagy (Jamart et al., 2012a,b; Martin-Rincon et al., 2017). The combination of a small reduction in muscle mass with a potentially lower mitochondrial density could have lowered the extraction and utilization of O_2_, thereby limiting VO_2_peak (Holmberg, 2015). Although, the mitochondrial respiratory capacity in the leg muscles is reduced after exercising extensively on several consecutive days (Boushel et al., 2015), leg muscles have excess mitochondrial respiration capacity (Boushel et al., 2011, 2015).

A reduction in VO_2_peak during whole-body exercise may be caused by lower oxygen delivery (Amann and Calbet, 2008) and/or central mechanisms of fatigue (Gandevia, 2001; Enoka and Duchateau, 2016; Torres-Peralta et al., 2016). Indeed, our overweight volunteers had not been exercising regularly and, therefore, they might have been more susceptible to central mechanisms of fatigue (Bachasson et al., 2016; O'Leary et al., 2017).

To further explore whether the protein supplementation could have facilitated the development of central fatigue the tryptophan ratio was calculated (Fernstrom and Wurtman, 1972; Fernstrom, 2013). Since neutral amino acids compete for transport across the brain blood barrier, this ratio determines the concentration of tryptophan in the central nervous system (Fernstrom and Wurtman, 1972). Since under normal conditions tryptophan hydroxylase, the rate-limiting enzyme in the conversion of tryptophan to serotonin, is only partially saturated with substrate, the rate of serotonin synthesis can be increased or decreased rapidly by raising or lowering brain tryptophan concentrations (Fernstrom, 2013). Although, elevated serotoninergic tone has been linked to central fatigue (Meeusen et al., 2006), our present findings are more compatible with a similar reduction of serotoninergic tone in both groups.

A reduction of the levels of catecholamine neurotransmitters (dopamine and noradrenaline) in the central nervous system could also contribute to central fatigue (Meeusen et al., 2006). Here, neither the plasma concentration of tyrosine nor the tyrosine ratio (data not shown) changed significantly from PRE to the end of CRE and CD, determining a likely similar tyrosine bioavailability to the central nervous system in both groups (Fernstrom, 2013). Thus, our amino acid data are compatible with a reduced serotoninergic and unchanged catecholaminergic and dopaminergic tone, which according to the current understanding should have attenuated central fatigue to a similar extent in both groups. Moreover, the fact that performance after PRE was reduced only during leg pedaling speaks against a major role for central fatigue, which should have also worsened arm-cranking performance. To further clarify the role played by central fatigue, specific neuromuscular tests will be required in future studies.

### Low intensity exercise helps to preserve lean mass in the extremities exercised during a severe energy deficit

In previous reports, aerobic or resistance exercise three to five times a week (15–60 min/session) during 16 weeks on a LCD (energy deficit of 4184 kJ/day) attenuated the loss of FFM by ~50% (Janssen and Ross, 1999; Rice et al., 1999; Janssen et al., 2002). In previous studies, no clear distinction between exercised and non-exercised muscles precluded to address the question of whether non-exercised muscles are also protected or if the exercise volume is also a factor determining the FFM-sparing effect of exercise during dieting (Chaston et al., 2007). In agreement with our current results, the combination of diet plus aerobic exercise reduced the loss of lean tissue (as assessed by magnetic resonance imaging) by ~28%, compared to diet alone (Ross et al., 1996). This value is similar to the lean mass sparing effect of 45 min arm cranking, but less than elicited by 8 h of walking in the leg muscles, observed here.

Observational dietary investigations with or without exercise report that exercise during LCD may attenuate the loss of lean tissue mass by 25–50% (Chaston et al., 2007). For more severe energy deficit, as during VLCD, previous studies indicate that exercise may reduce the loss of lean mass by 8–15% (Eston et al., 1992; Pronk et al., 1992; Hoie et al., 1993). However, the amount of exercise performed in previous studies was much lower than in the present investigation. However, the nature of the exercise stimulus is difficult to assess independently of diet and, particularly when comparing studies involving different diets and forms of exercise.

In the present investigation, even without ingestion of protein, exercise clearly preserved lean mass, as shown clearly by comparing the exercised and control arms. The extent of preservation was dependent on the volume of exercise as suggested by the greater sparing observed in the legs (8 h/d) than the exercised arms (45 min 7 d). However, alternative explanations are possible since legs and arms might have different proteolytic responses. In fact, we have recently shown that following 7 days of bed rest the muscle mass was reduced only in the legs, indicating that the legs might be more sensitive to proteolytic signals than the arms (Bienso et al., 2012; Guerra et al., 2014). Here, however, the legs lost proportionally less lean mass than the arms, further emphasizing the protective role of exercise on muscle mass, even during an extreme energy deficit. Nevertheless, these results should be interpreted with caution, since outcomes may depend on the training status of each muscle.

In agreement with our present observations, whole-body protein synthesis is reduced to a similar extent by starvation for 5 days and by consuming a VLCD (2.5 MJ/day, 18 g protein/day) for 9 days, both of which elicited a 5% loss of body weight (Afolabi et al., 2007). At the same time, protein oxidation was elevated only by starvation (Afolabi et al., 2007). Thus, muscle contraction facilitates nitrogen retention in the muscles exercised, even under severe energy deficit and regardless of whether protein is ingested or not.

### Protein supplementation during 4 days of severe energy deficit does not preserve more lean mass

Essential amino acids, and in particular leucine, are potent stimulators of protein synthesis (Blomstrand et al., 2006; Dreyer et al., 2008) and exercise may potentiate this anabolic response (Blomstrand and Saltin, 2001; Blomstrand et al., 2006; Dreyer et al., 2008). However, in contrast to our hypothesis, ingestion of proteins during the 4 days of severe energy deficit was not associated with greater preservation of lean mass. The whey protein ingested is highly soluble in aqueous solutions and rapidly absorbed in the intestines (Calbet and Holst, 2004), and known to efficiently stimulate protein synthesis and attenuate protein breakdown in humans (Phillips, 2011). Moreover, our subjects drank the protein solution within a 10-min period to elicit aminoacidemia rapidly, which stimulates protein synthesis more efficiently than repeated administration of smaller amounts of amino acids (West et al., 2011).

Although the amount of protein administered here (0.8 g/kg body weight/day) may be sufficient for maximal stimulation of protein synthesis in both rested and exercised muscles (Breen et al., 2011; Witard et al., 2014), a greater quantity may be required to achieve significant stimulation under conditions of severe energy deficit. Protein synthesis may be activated maximally after the ingestion of ~10 g of EAA containing about 2.1 g of leucine (Cuthbertson et al., 2005), with additional leucine or protein producing no greater stimulation (Glynn et al., 2010). Thus, the amount and type of protein administered in the present study should have been adequate to attenuate the loss of lean mass. In contrast, it has been reported that dietary protein levels 2-3-fold higher than the Recommended Dietary Allowance (RDA) for protein halve the loss of lean mass in subjects on a LCD (40% energy deficit; Pasiakos et al., 2013). However, a major difference between the study of Pasiakos et al. (2013) and the present investigation is the magnitude of the energy deficit and that we administered the protein alone, without carbohydrates. In fact, co-ingestion of proteins and carbohydrates may potentiate the insulin response to feeding (Calbet and MacLean, 2002; Allerton et al., 2016), although such co-ingestion during the recovery of healthy men from a single bout of endurance exercise does not result in more protein synthesis than ingestion of the same amount of protein alone (Howarth et al., 2009).

### Why protein supplementation did not result in greater preservation of lean mass?

The lack of any preservation of lean mass by ingestion of protein may reflect a similar catabolic response to CRE by both groups. The plasma level of leucine was increased in the group ingesting sucrose and since these subjects did not ingest any protein, this leucine must have originated from endogenous sources (Jensen et al., 1988). This is indicative of protein breakdown (Pozefsky et al., 1976; Umpleby et al., 1995) since leucine is an essential amino acid that cannot be synthesized and its oxidation is elevated during fasting (Nair et al., 1987). The higher plasma level of leucine in the protein group following CRE probably reflects the substantial amount of this amino acid ingested (about 9.5 g/day, in three individual doses of 3.2 g each).

In both groups testosterone was reduced while cortisol was increased, as previously reported in the adaptation to fasting (Nair et al., 1987; Cahill, 2006). However, the catabolic index (cortisol/free testosterone) tended to rise slightly more in PRO than the SU group. Interestingly, the loss of lean mass could be predicted from the change in the catabolic index.

Thus, it appears that when the energy deficit is severe (about 23 MJ/day in the present experiment), ingestion of protein results in little, if any, preservation of lean mass, since the protein breakdown elicited by the higher level of cortisol in combination with the reduced level of testosterone predominates over the anabolic effect by the increased serum leucine and BCAA. This conclusion agrees with the reduction in protein synthesis and leucine oxidation and rise in protein breakdown observed in obese volunteers following 3 weeks on a protein-free diet (only 2099 kJ/day in the form of glucose; Clugston and Garlick, 1982). Moreover, the increase of cortisol might have blunted the initiation of translation through the inhibition of 4E-BP1 phosphorylation (Shah et al., 2000).

In summary, our present findings demonstrate that during a severe energy deficit lasting a few days exercise results in remarkable preservation of lean mass. This effect does not require ingestion of proteins and can be attained with low-intensity exercise, such as walking or arm cranking. Although, a small volume of daily exercise can attenuate the loss of lean mass by about 30%, greater preservation can be achieved with longer sessions of exercise. During a severe energy deficit, administration of a protein solution without carbohydrates may cause a greater rise in the plasma level of cortisol and the catabolic index (cortisol/testosterone) than administration of carbohydrates alone. It remains to be determined whether a combination of carbohydrates and protein, larger amounts of protein and/or resistance exercise preserve lean mass more efficiently. Finally, we have demonstrated clearly that the lean mass preserving effect of exercise is constrained to the exercised muscles and may be different for the muscles of the upper and lower extremities in humans.

## Author contributions

Conception and design of the experiments: JC and HH; pre-testing, experimental preparation, data collection, and analysis: all co-authors. All co-authors read and approved the final version of the manuscript.

### Conflict of interest statement

The authors declare that the research was conducted in the absence of any commercial or financial relationships that could be construed as a potential conflict of interest.

## References

[B1] Achten J. Gleeson M. Jeukendrup A. E. (2002). Determination of the exercise intensity that elicits maximal fat oxidation. Med. Sci. Sports Exerc. 34, 92–97. 10.1097/00005768-200201000-00015 11782653

[B2] Afolabi P. R. Jahoor F. Jackson A. A. Stubbs J. Johnstone A. M. Faber P.. (2007). The effect of total starvation and very low energy diet in lean men on kinetics of whole body protein and five hepatic secretory proteins. Am. J. Physiol. Endocrinol. Metab. 293, E1580–E1589. 10.1152/ajpendo.00169.2007 17878226

[B3] Allerton D. M. Campbell M. D. Gonzalez J. T. Rumbold P. L. West D. J. Stevenson E. J. (2016). Co-ingestion of whey protein with a carbohydrate-rich breakfast does not affect glycemia, insulinemia or subjective appetite following a subsequent meal in healthy males. Nutrients 8:116. 10.3390/nu8030116 26927166 PMC4808846

[B4] Aloia J. F. Vaswani A. Ma R. Flaster E. (1995). To what extent is bone mass determined by fat-free or fat mass? Am. J. Clin. Nutr. 61, 1110–1114. 7733036 10.1093/ajcn/61.4.1110

[B5] Amann M. Calbet J. A. (2008). Convective oxygen transport and fatigue. J. Appl. Physiol. 104, 861–870. 10.1152/japplphysiol.01008.2007 17962570

[B6] Apro W. Blomstrand E. (2010). Influence of supplementation with branched-chain amino acids in combination with resistance exercise on p70S6 kinase phosphorylation in resting and exercising human skeletal muscle. Acta Physiol. 200, 237–248. 10.1111/j.1748-1716.2010.02151.x 20528801

[B7] Bachasson D. Decorte N. Wuyam B. Millet G. Y. Verges S. (2016). Original research: central and peripheral quadriceps fatigue in young and middle-aged untrained and endurance-trained men: a comparative study. Exp. Biol. Med. 241, 1844–1852. 10.1177/1535370216654225 27287015 PMC5027946

[B8] Bhasin S. Storer T. W. Berman N. Callegari C. Clevenger B. Phillips J.. (1996). The effects of supraphysiologic doses of testosterone on muscle size and strength in normal men. N. Engl. J. Med. 335, 1–7. 10.1056/NEJM199607043350101 8637535

[B9] Bienso R. S. Ringholm S. Kiilerich K. Aachmann-Andersen N. J. Krogh-Madsen R. Guerra B.. (2012). GLUT4 and glycogen synthase are key players in bed rest-induced insulin resistance. Diabetes 61, 1090–1099. 10.2337/db11-0884 22403297 PMC3331744

[B10] Blomstrand E. Saltin B. (2001). BCAA intake affects protein metabolism in muscle after but not during exercise in humans. Am. J. Physiol. Endocrinol. Metab. 281, E365–E374. 11440914 10.1152/ajpendo.2001.281.2.E365

[B11] Blomstrand E. Eliasson J. Karlsson H. K. Kohnke R. (2006). Branched-chain amino acids activate key enzymes in protein synthesis after physical exercise. J. Nutr. 136, 269S–273S. 16365096 10.1093/jn/136.1.269S

[B12] Boushel R. Gnaiger E. Calbet J. A. Gonzalez-Alonso J. Wright-Paradis C. Sondergaard H.. (2011). Muscle mitochondrial capacity exceeds maximal oxygen delivery in humans. Mitochondrion 11, 303–307. 10.1016/j.mito.2010.12.006 21147270

[B13] Boushel R. Gnaiger E. Larsen F. J. Helge J. W. Gonzalez-Alonso J. Ara I. (2015). Maintained peak leg and pulmonary VO despite substantial reduction in muscle mitochondrial capacity. Scand. J. Med. Sci. Sports 25(Suppl. 4), 135–143. 10.1111/sms.12613 26589127

[B14] Breen L. Philp A. Witard O. C. Jackman S. R. Selby A. Smith K.. (2011). The influence of carbohydrate-protein co-ingestion following endurance exercise on myofibrillar and mitochondrial protein synthesis. J. Physiol. 589, 4011–4025. 10.1113/jphysiol.2011.211888 21746787 PMC3179999

[B15] Cahill G. F. Jr. (2006). Fuel metabolism in starvation. Annu. Rev. Nutr. 26, 1–22. 10.1146/annurev.nutr.26.061505.111258 16848698

[B16] Calbet J. A. Holst J. J. (2004). Gastric emptying, gastric secretion and enterogastrone response after administration of milk proteins or their peptide hydrolysates in humans. Eur. J. Nutr. 43, 127–139. 10.1007/s00394-004-0448-4 15168035

[B17] Calbet J. A. MacLean D. A. (2002). Plasma glucagon and insulin responses depend on the rate of appearance of amino acids after ingestion of different protein solutions in humans. J. Nutr. 132, 2174–2182. 12163658 10.1093/jn/132.8.2174

[B18] Calbet J. A. Moysi J. S. Dorado C. Rodriguez L. P. (1998). Bone mineral content and density in professional tennis players. Calcif. Tissue Int. 62, 491–496. 10.1007/s002239900467 9576975

[B19] Calbet J. A. Ponce-Gonzalez J. G. Perez-Suarez I. J. de la Calle Herrero Holmberg H. C. (2015). A time-efficient reduction of fat mass in 4 days with exercise and caloric restriction. Scand. J. Med. Sci. Sports 25, 223–233. 10.1111/sms.12194 24602091

[B20] Chaston T. B. Dixon J. B. O'Brien P. E. (2007). Changes in fat-free mass during significant weight loss: a systematic review. Int. J. Obes. 31, 743–750. 10.1038/sj.ijo.0803483 17075583

[B21] Clugston G. A. Garlick P. J. (1982). The response of whole-body protein turnover to feeding in obese subjects given a protein-free, low-energy diet for three weeks. Hum. Nutr. Clin. Nutr. 36, 391–397. 7174360

[B22] Craig C. L. Marshall A. L. Sjöström M. Bauman A. E. Booth M. L. Ainsworth B. E.. (2003). International physical activity questionnaire: 12-country reliability and validity. Med. Sci. Sports Exerc. 35, 1381–1395. 10.1249/01.MSS.0000078924.61453.FB 12900694

[B23] Crowley M. A. Matt K. S. (1996). Hormonal regulation of skeletal muscle hypertrophy in rats: the testosterone to cortisol ratio. Eur. J. Appl. Physiol. Occup. Physiol. 73, 66–72. 10.1007/BF00262811 8861671

[B24] Cuthbertson D. Smith K. Babraj J. Leese G. Waddell T. Atherton P.. (2005). Anabolic signaling deficits underlie amino acid resistance of wasting, aging muscle. FASEB J. 19, 422–424. 10.1096/fj.04-2640fje 15596483

[B25] Dreyer H. C. Drummond M. J. Pennings B. Fujita S. Glynn E. L. Chinkes D. L.. (2008). Leucine-enriched essential amino acid and carbohydrate ingestion following resistance exercise enhances mTOR signaling and protein synthesis in human muscle. Am. J. Physiol. Endocrinol. Metab. 294, E392–E400. 10.1152/ajpendo.00582.2007 18056791 PMC2706121

[B26] Enoka R. M. Duchateau J. (2016). Translating fatigue to human performance. Med. Sci. Sports Exerc. 48, 2228–2238. 10.1249/MSS.0000000000000929 27015386 PMC5035715

[B27] Eston R. G. Shephard S. Kreitzman S. Coxon A. Brodie D. A. Lamb K. L.. (1992). Effect of very low calorie diet on body composition and exercise response in sedentary women. Eur. J. Appl. Physiol. Occup. Physiol. 65, 452–458. 10.1007/BF00243513 1425652

[B28] Fernstrom J. D. (2013). Large neutral amino acids: dietary effects on brain neurochemistry and function. Amino Acids 45, 419–430. 10.1007/s00726-012-1330-y 22677921

[B29] Fernstrom J. D. Wurtman R. J. (1972). Brain serotonin content: physiological regulation by plasma neutral amino acids. Science 178, 414–416. 10.1126/science.178.4059.414 5077329

[B30] Fernstrom M. Bakkman L. Tonkonogi M. Shabalina I. G. Rozhdestvenskaya Z. Mattsson C. M.. (2007). Reduced efficiency, but increased fat oxidation, in mitochondria from human skeletal muscle after 24-h ultraendurance exercise. J. Appl. Physiol. 102, 1844–1849. 10.1152/japplphysiol.01173.2006 17234801

[B31] Gandevia S. C. (2001). Spinal and supraspinal factors in human muscle fatigue. Physiol. Rev. 81, 1725–1789. 11581501 10.1152/physrev.2001.81.4.1725

[B32] Glynn E. L. Fry C. S. Drummond M. J. Dreyer H. C. Dhanani S. Volpi E.. (2010). Muscle protein breakdown has a minor role in the protein anabolic response to essential amino acid and carbohydrate intake following resistance exercise. Am. J. Physiol. Regul. Integr. Comp. Physiol. 299, R533–R540. 10.1152/ajpregu.00077.2010 20519362 PMC2928613

[B33] Guerra B. Ponce-Gonzalez J. G. Morales-Alamo D. Guadalupe-Grau A. Kiilerich K. Fuentes T.. (2014). Leptin signaling in skeletal muscle after bed rest in healthy humans. Eur. J. Appl. Physiol. 114, 345–357. 10.1007/s00421-013-2779-4 24292882

[B34] Guezennec C. Y. Satabin P. Legrand H. Bigard A. X. (1994). Physical performance and metabolic changes induced by combined prolonged exercise and different energy intakes in humans. Eur. J. Appl. Physiol. Occup. Physiol. 68, 525–530. 10.1007/BF00599524 7957146

[B35] Harger-Domitrovich S. G. McClaughry A. E. Gaskill S. E. Ruby B. C. (2007). Exogenous carbohydrate spares muscle glycogen in men and women during 10 h of exercise. Med. Sci. Sports Exerc. 39, 2171–2179. 10.1249/mss.0b013e318157a650 18046188

[B36] Hoie L. H. Bruusgaard D. Thom E. (1993). Reduction of body mass and change in body composition on a very low calorie diet. Int. J. Obes. Relat. Metab. Disord. 17, 17–20. 8383636

[B37] Holmberg H. C. (2015). The elite cross-country skier provides unique insights into human exercise physiology. Scand. J. Med. Sci. Sports 25(Suppl. 4), 100–109. 10.1111/sms.12601 26589123

[B38] Howarth K. R. Moreau N. A. Phillips S. M. Gibala M. J. (2009). Coingestion of protein with carbohydrate during recovery from endurance exercise stimulates skeletal muscle protein synthesis in humans. J. Appl. Physiol. 106, 1394–1402. 10.1152/japplphysiol.90333.2008 19036894

[B39] Jamart C. Benoit N. Raymackers J. M. Kim H. J. Kim C. K. Francaux M. (2012a). Autophagy-related and autophagy-regulatory genes are induced in human muscle after ultraendurance exercise. Eur. J. Appl. Physiol. 112, 3173–3177. 10.1007/s00421-011-2287-3 22194006

[B40] Jamart C. Francaux M. Millet G. Y. Deldicque L. Frere D. Feasson L. (2012b). Modulation of autophagy and ubiquitin-proteasome pathways during ultra-endurance running. J. Appl. Physiol. 112, 1529–1537. 10.1152/japplphysiol.00952.2011 22345427

[B41] Janssen I. Fortier A. Hudson R. Ross R. (2002). Effects of an energy-restrictive diet with or without exercise on abdominal fat, intermuscular fat, and metabolic risk factors in obese women. Diabetes Care 25, 431–438. 10.2337/diacare.25.3.431 11874926

[B42] Janssen I. Ross R. (1999). Effects of sex on the change in visceral, subcutaneous adipose tissue and skeletal muscle in response to weight loss. Int. J. Obes. Relat. Metab. Disord. 23, 1035–1046. 10.1038/sj.ijo.0801038 10557024

[B43] Jensen M. D. Miles J. M. Gerich J. E. Cryer P. E. Haymond M. W. (1988). Preservation of insulin effects on glucose production and proteolysis during fasting. Am. J. Physiol. 254, E700–E707. 3287951 10.1152/ajpendo.1988.254.6.E700

[B44] Johnston C. S. Tjonn S. L. Swan P. D. (2004). High-protein, low-fat diets are effective for weight loss and favorably alter biomarkers in healthy adults. J. Nutr. 134, 586–591. 14988451 10.1093/jn/134.3.586

[B45] Kimber N. E. Ross J. J. Mason S. L. Speedy D. B. (2002). Energy balance during an ironman triathlon in male and female triathletes. Int. J. Sport Nutr. Exerc. Metab. 12, 47–62. 10.1123/ijsnem.12.1.47 11993622

[B46] Krieger J. W. Sitren H. S. Daniels M. J. Langkamp-Henken B. (2006). Effects of variation in protein and carbohydrate intake on body mass and composition during energy restriction: a meta-regression. Am. J. Clin. Nutr. 83, 260–274. 16469983 10.1093/ajcn/83.2.260

[B47] Krug A. L. Macedo A. G. Zago A. S. Rush J. W. Santos C. F. Amaral S. L. (2016). High-intensity resistance training attenuates dexamethasone-induced muscle atrophy. Muscle Nerve 53, 779–788. 10.1002/mus.24906 26355638

[B48] Longland T. M. Oikawa S. Y. Mitchell C. J. Devries M. C. Phillips S. M. (2016). Higher compared with lower dietary protein during an energy deficit combined with intense exercise promotes greater lean mass gain and fat mass loss: a randomized trial. Am. J. Clin. Nutr. 103, 738–746. 10.3945/ajcn.115.119339 26817506

[B49] Martin-Rincon M. Morales-Alamo D. Calbet J. A. L. (2017). Exercise-mediated modulation of autophagy in skeletal muscle. Scand. J. Med. Sci. Sports [Epub ahead of print]. 10.1111/sms.12945 28685860

[B50] Marks B. L. Rippe J. M. (1996). The importance of fat free mass maintenance in weight loss programmes. Sports Med. 22, 273–281. 10.2165/00007256-199622050-00001 8923645

[B51] Meeusen R. Watson P. Hasegawa H. Roelands B. Piacentini M. F. (2006). Central fatigue: the serotonin hypothesis and beyond. Sports Med. 36, 881–909. 10.2165/00007256-200636100-00006 17004850

[B52] Mettler S. Mitchell N. Tipton K. D. (2010). Increased protein intake reduces lean body mass loss during weight loss in athletes. Med. Sci. Sports Exerc. 42, 326–337. 10.1249/MSS.0b013e3181b2ef8e 19927027

[B53] Muller M. J. Bosy-Westphal A. Kutzner D. Heller M. (2002). Metabolically active components of fat-free mass and resting energy expenditure in humans: recent lessons from imaging technologies. Obes. Rev. 3, 113–122. 10.1046/j.1467-789X.2002.00057.x 12120418

[B54] Nair K. S. Woolf P. D. Welle S. L. Matthews D. E. (1987). Leucine, glucose, and energy metabolism after 3 days of fasting in healthy human subjects. Am. J. Clin. Nutr. 46, 557–562. 3661473 10.1093/ajcn/46.4.557

[B55] O'Leary T. J. Collett J. Howells K. Morris M. G. (2017). Endurance capacity and neuromuscular fatigue following high- vs moderate-intensity endurance training: a randomized trial. Scand. J. Med. Sci. Sports. [Epub ahead of print]. 10.1111/sms.12854 28207951

[B56] Opstad P. K. (2001). Endocrine and metabolic changes during exhaustive multifactorial military stress. results from studies during the ranger training course of the Norwegian Military Academy, in The Effect of Prolonged Military Activities in Man. Physiological and Biochemical Changes. Possible Means of Rapid Recuperation. RTO Meeting Proceedings 42 (Oslo: RTO/NATO).

[B57] Pasiakos S. M. Cao J. J. Margolis L. M. Sauter E. R. Whigham L. D. McClung J. P.. (2013). Effects of high-protein diets on fat-free mass and muscle protein synthesis following weight loss: a randomized controlled trial. FASEB J. 27, 3837–3847. 10.1096/fj.13-230227 23739654

[B58] Phillips S. M. (2011). The science of muscle hypertrophy: making dietary protein count. Proc. Nutr. Soc. 70, 100–103. 10.1017/S002966511000399X 21092368

[B59] Ponce-Gonzalez J. G. Rodriguez-Garcia L. Losa-Reyna J. Guadalupe-Grau A. Rodriguez-Gonzalez F. G. Diaz-Chico B. N. (2016). Androgen receptor gene polymorphisms influence fat accumulation: a longitudinal study from adolescence to adult age. Scand. J. Med. Sci. Sports 26, 1313–1320. 10.1111/sms.12587 26634957

[B60] Pozefsky T. Tancredi R. G. Moxley R. T. Dupre J. Tobin J. D. (1976). Effects of brief starvation on muscle amino acid metabolism in nonobese man. J. Clin. Invest. 57, 444–449. 10.1172/JCI108295 1254728 PMC436668

[B61] Pronk N. P. Donnelly J. E. Pronk S. J. (1992). Strength changes induced by extreme dieting and exercise in severely obese females. J. Am. Coll. Nutr. 11, 152–158. 1578090

[B62] Rice B. Janssen I. Hudson R. Ross R. (1999). Effects of aerobic or resistance exercise and/or diet on glucose tolerance and plasma insulin levels in obese men. Diabetes Care 22, 684–691. 10.2337/diacare.22.5.684 10332666

[B63] Ross R. Rissanen J. Pedwell H. Clifford J. Shragge P. (1996). Influence of diet and exercise on skeletal muscle and visceral adipose tissue in men. J. Appl. Physiol. 81, 2445–2455. 9018491 10.1152/jappl.1996.81.6.2445

[B64] Sabapathy S. Morris N. R. Schneider D. A. (2006). Ventilatory and gas-exchange responses to incremental exercise performed with reduced muscle glycogen content. J. Sci. Med. Sport 9, 267–273. 10.1016/j.jsams.2006.03.024 16682251

[B65] Sahlin K. Shabalina I. G. Mattsson C. M. Bakkman L. Fernstrom M. Rozhdestvenskaya Z.. (2010). Ultraendurance exercise increases the production of reactive oxygen species in isolated mitochondria from human skeletal muscle. J. Appl. Physiol. 108, 780–787. 10.1152/japplphysiol.00966.2009 20110545 PMC2853199

[B66] Serrano-Sanchez J. A. Delgado-Guerra S. Olmedillas H. Guadalupe-Grau A. Arteaga-Ortiz R. Sanchis-Moysi J.. (2010). Adiposity and age explain most of the association between physical activity and fitness in physically active men. PLoS ONE 5:e13435. 10.1371/journal.pone.0013435 20976154 PMC2956676

[B67] Shah O. J. Kimball S. R. Jefferson L. S. (2000). Acute attenuation of translation initiation and protein synthesis by glucocorticoids in skeletal muscle. Am. J. Physiol. Endocrinol. Metab. 278, E76–E82. 10644539 10.1152/ajpendo.2000.278.1.E76

[B68] Shaw K. A. Gennat H. C. O'Rourke P. Del Mar C. (2009). Exercise for overweight or obesity. Cochrane Database Syst. Rev. 4:CD003817 10.1002/14651858.CD003817.pub3 PMC9017288 17054187

[B69] Snijders T. Res P. T. Smeets J. S. van Vliet S. van Kranenburg J. Maase K.. (2015). Protein ingestion before sleep increases muscle mass and strength gains during prolonged resistance-type exercise training in healthy young men. J. Nutr. 145, 1178–1184. 10.3945/jn.114.208371 25926415

[B70] Torres-Peralta R. Morales-Alamo D. Gonzalez-Izal M. Losa-Reyna J. Perez-Suarez I. Izquierdo M. (2016). Task failure during exercise to exhaustion in normoxia and hypoxia is due to reduced muscle activation caused by central mechanisms while muscle metaboreflex does not limit performance. Front. Physiol. 6:414 10.3389/fphys.2015.00414 26793117 PMC4707284

[B71] Umpleby A. M. Scobie I. N. Boroujerdi M. A. Sonksen P. H. (1995). The effect of starvation on leucine, alanine and glucose metabolism in obese subjects. Eur. J. Clin. Invest. 25, 619–626. 10.1111/j.1365-2362.1995.tb01755.x 7589020

[B72] Van Hall G. Jensen-Urstad M. Rosdahl H. Holmberg H. C. Saltin B. Calbet J. A. (2003). Leg and arm lactate and substrate kinetics during exercise. Am. J. Physiol. Endocrinol. Metab. 284, E193–E205. 10.1152/ajpendo.00273.2002 12388120

[B73] Vicente-Rodriguez G. Ara I. Perez-Gomez J. Dorado C. Calbet J. A. (2005). Muscular development and physical activity as major determinants of femoral bone mass acquisition during growth. Br. J. Sports Med. 39, 611–616. 10.1136/bjsm.2004.014431 16118297 PMC1725300

[B74] Vogels N. Westerterp-Plantenga M. S. (2007). Successful long-term weight maintenance: a 2-year follow-up. Obesity 15, 1258–1266. 10.1038/oby.2007.147 17495202

[B75] West D. W. Burd N. A. Coffey V. G. Baker S. K. Burke L. M. Hawley J. A.. (2011). Rapid aminoacidemia enhances myofibrillar protein synthesis and anabolic intramuscular signaling responses after resistance exercise. Am. J. Clin. Nutr. 94, 795–803. 10.3945/ajcn.111.013722 21795443

[B76] Witard O. C. Jackman S. R. Breen L. Smith K. Selby A. Tipton K. D. (2014). Myofibrillar muscle protein synthesis rates subsequent to a meal in response to increasing doses of whey protein at rest and after resistance exercise. Am. J. Clin. Nutr. 99, 86–95. 10.3945/ajcn.112.055517 24257722

[B77] Zinner C. Morales-Alamo D. Ortenblad N. Larsen F. J. Schiffer T. A. Willis S. J.. (2016). The physiological mechanisms of performance enhancement with sprint interval training differ between the upper and lower extremities in humans. Front. Physiol. 7:426. 10.3389/fphys.2016.00426 27746738 PMC5043010

